# Vinegar as a Post Partum Hemostatic

**Published:** 1879-12

**Authors:** 


### VINEGAR AS A POST PARTUM HEMOSTATIC.

At a meeting of the American Gynecological Society, Dr. Penrose, in a paper on vinegar as a remedy in the treatment of post partum hemorrhage, presented the following advantages:

1. It could be easily obtained.
2. It could be easily applied, and instantly, without special apparatus.
3. It always cured the hemorrhage, at least it had not failed in his practice.
4. It was sufficiently irritating to excite the most sluggish uterus to contraction, and yet not so irritating as to be subsequently injurious.
5. It was an admirable antiseptic.
6. It acted on the lining membrane of the uterus as an astringent.

The remedy was applied as follows: saturate a rag with vinegar, carry it into the cavity of the uterus and squeeze it.

In the vast majority of cases the hemorrhage ceased as if by magic, when the vinegar passed over the surface of the uterus and vagina. It could be easily repeated if the first application failed.—*Cincinnati Medical News.*

